# Supplementary material for: Nonlinearity of the post-spinel transition and its expression in slabs and plumes worldwide
Source: Nat Commun. 2025 Jan 26;16:1039. doi: 10.1038/s41467-025-56231-z (PMC11762276; doi:10.1038/s41467-025-56231-z)
Supplement: Supplementary file 1 — Supplementary Information [file 41467_2025_56231_MOESM1_ESM.pdf]

Supporting Information for

Nonlinearity of the post-spinel transition and its expression in slabs and plumes worldwide

Junjie Dong[1,2,6,*], Rebecca A. Fischer[1], Lars Stixrude[3], Matthew C. Brennan[1,7], Kierstin Daviau[1,8], Terry-Ann Suer[1,9], Katlyn M. Turner[1,10], Yue Meng[4], Vitali B. Prakapenka[5].

[1] Department of Earth and Planetary Sciences, Harvard University, Cambridge, Massachusetts, United States of America.
[2] Department of the History of Science, Harvard University, Cambridge, Massachusetts, United States of America.
[3] Department of Earth, Planetary, and Space Sciences, University of California, Los Angeles, California, United States of America.
[4] High Pressure Collaborative Access Team (HPCAT), X-Ray Science Division, Argonne National Laboratory, Argonne, Illinois, United States of America.
[5] Center for Advanced Radiation Sources, University of Chicago, Chicago, Illinois, United States of America.
—Affiliation listed above—
—Current address provided below—
[6] Now at Division of Geological and Planetary Sciences, California Institute of Technology, Pasadena, California, United States of America.
[7] Now at Shock and Detonation Physics Group, Los Alamos National Laboratory, Los Alamos, New Mexico, United States of America.
[8]Now at Toi-Ohomai Institute of Technology, Tauranga, New Zealand and School of Science, University of Waikato, Tauranga, New Zealand.
[9]Now at Laboratory for Laser Energetics, University of Rochester, Rochester, New York, United States of America.
[10]Now at MIT Media Lab, Massachusetts Institute of Technology, Cambridge, Massachusetts, United States of America.

[*]Corresponding Author: Junjie Dong (E-mail: dong2j@caltech.edu).

This PDF file includes:

Supporting text
Figs. S1 to S8
SI References

Supporting Information Text

Thermodynamic Modeling for the Post-Spinel Transitions in Mg_2SiO_4, $(Mg_{0.9}Fe_{0.1})_2SiO_4$, and Pyrolite

We use the Clapeyron slope of the post-spinel transition in Mg_2SiO_4 as a proxy for that in the mantle of pyrolite composition between 1400–2150 K. This is because the Clapeyron slopes for the post-spinel transition in these two compositions are nearly identical over this temperature range.

To support this view, we have modeled the post-spinel transitions in Mg_2SiO_4, $(Mg_{0.9}Fe_{0.1})_2SiO_4$, and pyrolite using the thermodynamic code HeFESTo (1, 2). In Figure S1, we demonstrate that the slope of the post-spinel transition is the same in these three compositions, and that the P–T location of the transitions differ slightly by <0.5 GPa; the binary loop of the post-spinel transitions in $(Mg_{0.9}Fe_{0.1})_2SiO_4$ and pyrolite is too narrow to be significant. The only notable difference is in the pyrolite composition, where *rw* would break into *gt* and *fp* above 2150 K, a transition not observed in Mg_2SiO_4 and $(Mg_{0.9}Fe_{0.1})_2SiO_4$. The *rw* ↔ *gt* + *fp* transition has been considered for the plumes in Figure 3, represented by the circles with hatched black lines.

Although the thermodynamic models are heuristic in investigating whether there is a significant difference in slopes between the analogue composition, Mg_2SiO_4, and the realistic pyrolite composition, it does not provide information on the exact magnitude of the Clapeyron slope due to unreliable extrapolations of entropy (ΔS) and volume (ΔV). For example, ΔS used in the thermodynamic model is not experimentally accessible and has been extrapolated from the low-temperature data (<500 K) on heat capacity without high-temperature validation. Kojitani et al. (2016) (3) demonstrated that minor adjustments in certain thermodynamic parameters, including heat capacity, can significantly alter the predicted ΔS and hence the slope of the post-spinel phase transition, highlighting the unreliability of the thermodynamic models for accurate phase boundary predictions.

Here in particular, we note that these HeFESTo calculations use an old set of parameters—as in our new data have not been taken into account. HeFESTo imposes a linear boundary on Mg_2SiO_4 when inverting the reference entropy. This is why it looks different from the nonlinear post-spinel boundary we find directly from the phase stability observations. We also examined the confusion matrices and found that our logistic inversion of direct experimental observations provides higher classification accuracy compared to the HeFESTo prediction at the post-spinel boundary. (Figure S8). Therefore, instead of thermodynamic calculations, direct experimental observations of phase stability and phase boundaries remain the most effective way to determine the slope of the post-spinel transition at high P–T conditions.

Details of High Pressure–Temperature Experimental Methods

High-pressure conditions were generated using diamond anvils with 300 µm culets mounted within short symmetric cells (samples D2, D5, D6, D10, D13, D14) or a gas-membrane cell (samples D1m, D4m, D11m). The Re gaskets were pre-indented to pressures of 20–28 GPa based on ruby fluorescence and diamond Raman spectroscopies (4, 5). A sample chamber (120 µm diameter) was then drilled into each pre-indented Re gasket.

The starting materials consisted of synthetic forsterite Mg_2SiO_4 powder (99% purity, Alfa Aesar) that was thoroughly mixed with W powder (Puratronic, 99.999% purity, Alfa Aesar) in a roughly 3:1 ratio by volume using a mechanical ball mill. Energy-dispersive X-ray spectroscopy (EDS) was used to confirm the uniformity and exact composition of the mixture. Small amounts of this powder mixture were then pressed into flakes (~10 µm thick) and loaded into the sample chamber between two layers of pressed KCl. The KCl powder was dried in an oven for at least 6 hours before loading. To eliminate residual moisture, after loading the sample but before closing the cell, each sample was dried in a vacuum oven at 393–413 K (120–140 °C) for 1–7 hours.

Two identical Yb-doped CW fiber lasers (λ = 1064 nm) heated the sample from both sides during the experiment. At GESCARS, the X-ray beam was monochromatized to a wavelength of 0.3344 Å and focused onto areas of 2 µm × 3.5 µm, 2.5 µm × 3.5 µm, and 3 µm × 4 µm during three beamtimes in 2018-2019. At HPCAT, it was tuned to a wavelength of 0.4066 Å and focused onto an area of 2.9 µm × 4.8 µm during one beamtime in 2019. X-ray exposure times ranged from 3 to 60 seconds, and diffraction images were later integrated into 1D patterns using Dioptas (6).

In a typical experiment with the short symmetric cell (Figure S2), we first compressed the sample to a target pressure (usually 16–22 GPa) and then pre-heated the sample to 1200–1500 K until the diffraction peaks of either *wd* or *rw* became clear. After that, we began the heating cycle by increasing the temperature slowly while periodically collecting diffraction patterns. Three of our experiments were conducted in a gas-membrane cell. They were first heated to 2000–2200 K at some pressure below 20–21 GPa, and then very small incremental loads were applied from a gas membrane system to increase the pressure remotely while the sample was being heated (Figure S2).

Sample temperatures (T_{meas}) were measured spectroradiometrically, and the temperature of the KCl layers (T_{KCl}) for each data point were estimated as the mean of the sample temperature (T_{meas}) and the temperature of the diamond anvil culets, ~295 K (7). In applying the approach of Campbell et al. for estimating the effective temperature of a pressure medium (7), we note that the equation in their study contains a typo, and the correct formula to calculate T_{KCl} should be written as follows: $T_{KCl} = \frac{3T_{meas}+295}{4} \pm \frac{T_{meas}-295}{4}$. Sample pressures P_W and P_{KCl} were determined using the equation of state (EoS) of W with T_{meas} (8) and the EoS of KCl with T_{KCl} (9), respectively. These two pressure scales have been cross-calibrated and should therefore be internally consistent. However, P_W deviates from P_{KCl} significantly at high temperatures, likely due to the lack of thermal EoS data for W above ~1673 K (8, 10). Therefore, we chose KCl as our primary pressure standard, and all pressures reported in this study are based on the EoS of KCl from (9). The Chebyshev series fit with the NumPy Python package (11)

was used to identify and subtract backgrounds from the integrated XRD patterns. Peaks from W and KCl were identified and fitted with Gaussian, Lorentzian, or Voigt functions using the LMFIT Python package (12, 13). The corresponding 2θ values were extracted and used to calculate the unit cell parameters of W and KCl. The diffraction lines used were typically (110), (200), (211), (220), (310), and (321) for W; and (110), (200), (211), and (220) for KCl. These diffraction lines, their precision, and their propagated errors in pressure are reported, along with the estimated pressure for each data point, in Figure S2 and Dataset S1.

Multi-Class Logistic Regression and Supervised Learning Algorithms and Their Applicability to Phase Diagram Determination

To accurately pinpoint a phase boundary's location from experimental data, we ideally need phase stability observations from both sides of the boundary. However, obtaining these experimental brackets becomes more challenging at higher pressures. Moreover, results from different runs or different labs may be inconsistent. Historically, most researchers have sketched phase boundaries by hand based on their own experimental data, without rigorously considering inconsistencies among different datasets. This approach fails to maximally utilize the information from all available experimental constraints, even though these high-pressure experiments are costly and time-intensive. It also fails to provide a comprehensive assessment of the dataset's reliability and accuracy.

In this context, we introduce a machine learning framework for a thorough analysis of phase stability observations when there are multiple stable phases or phase assemblage fields. This methodology combines multi-class logistic regression and supervised learning. Here, we applied our method to the global analysis of the Mg_2SiO_4 phase diagram data, with the aim of showcasing how our methodology can evaluate, select, and estimate the uncertainties associated with each phase boundary in a phase diagram.

To build a statistical model to describe the stability fields of a high-pressure phase diagram, the response variable (stable phase) must be categorical instead of numerical, and hence determining the location of a phase boundary should be considered a "classification problem" (14–16) since it involves assigning the observations to one stable mineral phase (discrete and categorical response) at a specific P–T condition (numerical predictor). Kavner et al. (2011) pioneered a similar but much simpler analysis of phase stability observations through binary logistic regression and tested it on the melting curve of platinum (15). However, their implementation 1) was limited to two stable phases with one phase boundary, 2) required additional assumptions regarding the shape of the phase boundary, and 3) did not include necessary procedures to prevent overfitting. For the Mg_2SiO_4 phase diagram, with three stability fields (wd, rw, and $bm + pe$) at transition zone conditions, we had to generalize the simple binary logistic model and estimate the probabilities of observing multiple stable phases simultaneously ($\hat{p}(Y|P,T)$ with $Y = k$, where k can be wd, rw, or $bm + pe$) at a given P–T condition. In the multi-class logistic model we propose here, the scenario that Y belongs (or does not belong) to one specific phase stability field k can be described as:

$$\hat{p}(Y|P,T) = \begin{cases} 0 & \text{, if } Y = k \\ 1 & \text{, if } Y \neq k \end{cases} \quad [1]$$

To describe the probability between 0 and 1, we can write the probability of observing phase $Y = k$ at a given P–T condition ($\hat{p}(Y = k|P,T)$) as a logistic function ($\frac{e^{f(X)}}{1+e^{f(X)}}$, where $f(X)$ is a nth degree polynomial function with two variables, $f(P,T)$):

$$\hat{p}(Y = k|P,T) = \frac{e^{\sum_{i,j=0}^{n} \beta_{i,j}^{k} P^i T^j}}{1 + e^{\sum_{i,j=0}^{n} \beta_{i,j}^{k} P^i T^j}} \quad [2]$$

Alternatively, Eq. 2 can be rewritten as:

$$\ln \frac{\hat{p}(Y = k|P,T)}{1 - \hat{p}(Y = k|P,T)} = \ln \frac{\hat{p}(Y = k|P,T)}{\hat{p}(Y \neq k|P,T)} = \sum_{i,j=0}^{n} \beta_{i,j}^{k} P^i T^j = f(P,T) \quad [3]$$

The quantities $\ln \frac{\hat{p}(Y=k|P,T)}{\hat{p}(Y \neq k|P,T)}$ and $\ln \frac{\hat{p}(Y=k|P,T)}{1-\hat{p}(Y=k|P,T)}$ are called log-odds and logit, respectively. We can interpret the logistic model as fitting the log-odds or logit as a polynomial function of P and T, $f(P,T)$.

We can then convert probability estimates from three separate models (where $k = wd$, rw, or $bm + pe$; $K = 3$) to one set of probability estimates using a multi-class generalization of the logistic function, which is also known as the normalized exponential or the *softmax* function:

$$\hat{p}(Y = k|P,T) = \frac{e^{\sum_{i,j=0}^{n} \beta_{i,j}^{k} P^i T^j}}{\sum_{h=1}^{K} e^{\sum_{i,j=0}^{n} \beta_{i,j}^{h} P^i T^j}} \quad [4]$$

The rescaled probability estimates add up to 1. We assign the stable phase to be the class with the highest probability, and the triple point is taken to be the P–T condition at which $\hat{p}(Y = wd) = \hat{p}(Y = rw) = \hat{p}(Y = bm + pe) = \frac{1}{3}$. The coefficients $\beta_{i,j}^{k}$ are estimated by minimizing a combined negative log-likelihood function, or total cross entropy, $-L$ (14):

$$-L = -\frac{1}{M} \sum_{m=1}^{M} \sum_{k=1}^{K} \{t_{m,k}(y_m = k) \cdot \ln [p_m(y_m = k)] + t_{m,l}(y \neq k) \cdot \ln [1 - p_m(y_m \neq k)]\} \quad [5]$$

where $t_{m,l}(y_i = k)$ is 1 if and only if the observation m belongs to phase k , $p_m(y_m = k)$ is the output probability that the observation m belongs to phase k, and M is the total number of observations. Unlike the binary case (15), the multi-class logistic model does not produce a simple, easy-to-interpret analytic solution; therefore, the phase boundaries between the three phases and their triple point for Mg_2SiO_4 were determined numerically using the Python package "scikit-learn" (17). A comprehensive review of the multi-class logistic model can be found in (16).

In the multi-class logistic model, a nonlinear phase boundary can be implemented in the log-odds/logit function (Equation 3), which is mathematically simplified as being a polynomial function of P and T. Ideally, a phase boundary should be described as a function of the thermoelastic properties of the phases involved, such as thermal expansion coefficient, entropy, heat capacity, etc., which can then be converted into a function of pressure, temperature, and chemical composition. However, it would be cumbersome to obtain an analytic approximation of these thermodynamic functions because such conversion would require solving a number of inexplicit functions in the EoS for each phase. For simplicity, we used polynomials in the log-odds/logit function (Equation 3) to capture the nonlinearity of the phase boundary without the mathematical burdens of extracting a complex analytic solution directly from physical thermodynamics. The high degree of freedom we introduce here into a conventional logistic model would allow the model to capture not only the location of the phase boundary, but also the noise in the experimental dataset. As the degree of the log-odds/logit polynomial keeps increasing, we would begin to fit a squiggly phase boundary that is consistent with more data points, even though only a smooth curve is thermodynamically expected. In contrast, our proposed machine learning framework is a robust way to identify the shape of a phase boundary and to estimate its slope. We applied a set of supervised learning algorithms from common machine learning practices to avoid overfitting, accompanied by regularization and bootstrapping.

We used supervised learning to constrain the multi-class logistic model for Mg_2SiO_4 phase boundaries (Figure S5). We first divided the compiled data, with 70% of the data going into a train set and 30% into a test set. Evaluating the model on both of these sets of data was crucial to avoid overfitting, because the model can otherwise overfit the train set to predict perfect responses but fail to perform well in the unseen test set.

Our approach was to describe the log-odds using polynomials of P and T from degree 1 to 10. However, overfitting could occur if we selected a high degree polynomial for the log-odds, and thus we applied a regularization method called *Lasso* or L_1 regularization. This regularization method constrains or regularizes the estimated coefficients (β_j) by modifying the least-squares loss function ($L(\beta)$) into a regularized loss function: $L_{Lasso}(\beta) = L(\beta) + \lambda \sum_{i,j=1}^{n} |\beta_{i,j}|$, where λ is a scaler that assigns weights to the regularization term and $\lambda \sum_{i,j=1}^{n} |\beta_{i,j}|$ is the regularization strength. We then used λ to discourage/penalize extreme values of β_j to avoid overfitting: when λ is sufficiently large, the regularized loss function $L_{Lasso}(\beta)$ becomes increasingly sensitive to $\lambda \sum_{i,j=1}^{n} |\beta_{i,j}|$. In such a scenario, a successful convergence would shrink β_{Lasso} to zero, or close to zero.

We optimized the hyperparameter C (the inverse of regularization strength, $\lambda = \frac{1}{C}$), along with other parameters in the scikit-learn package such as "multi_class" and "solver". Parameter tuning was achieved through a grid search with k-fold cross-validation, which involved further splitting the train set into k folds (k = 3–5). We then trained the model using k–1 folds, validating it on the remaining fold. The final evaluation of the parameters was based on the average over the k folds.

For model evaluation, we used a combination of two metrics: precision ($\frac{\text{true positive}}{\text{true positive + false positive}}$) and recall ($\frac{\text{true positive}}{\text{true positive + false negative}}$), which measure the proportion of accurate positive predictions and the ability to find all of the positive samples, respectively. Since precision and recall are inversely related, we used their harmonic mean, known as the F_1 score ($2 \times \frac{\text{precision} \times \text{recall}}{\text{precision+recall}}$), as our evaluation metric to maximize both precision and recall simultaneously. This score helps maintain a balance between precision and recall, with a high score indicating a more accurate model (Figure S6).

We then fitted the tuned model to the train and test sets. The log-odds/logit polynomial degree with the highest F_1 score on the test set was selected as the best degree. Lastly, we utilized the multi-class logistic regression with the chosen log-odds/logit polynomial degree to fit the entire dataset (the recombined train and test set) and obtained an optimized phase diagram (Figure S7).

To construct the phase diagram, we used logistic regression, which is a well-suited model for this study due to its simplicity and interpretability. While more complex classification algorithms such as Support Vector Machines (SVMs) and XGBoost are able to model nonlinear boundaries using kernel functions or decision tree ensembles, they often produce unrealistic oscillations in the phase boundaries. This behavior is particularly problematic when working with limited or noisy experimental data, as these classifiers often overfit the data to produce frequent sign reversals in the phase boundary slope—such behavior contradicts the thermodynamic expectation for solid-solid phase transitions. In contrast, the result of the logistic classifier is more physically consistent with the gradual, non-reversing changes in slope characteristic of solid-solid phase transitions. Details on the basics and differences of several common classification algorithms can be found in general machine learning resources such as "scikit-learn" (17) as well as geochemistry-specific machine learning resources such as "Geochemistry π" (18).

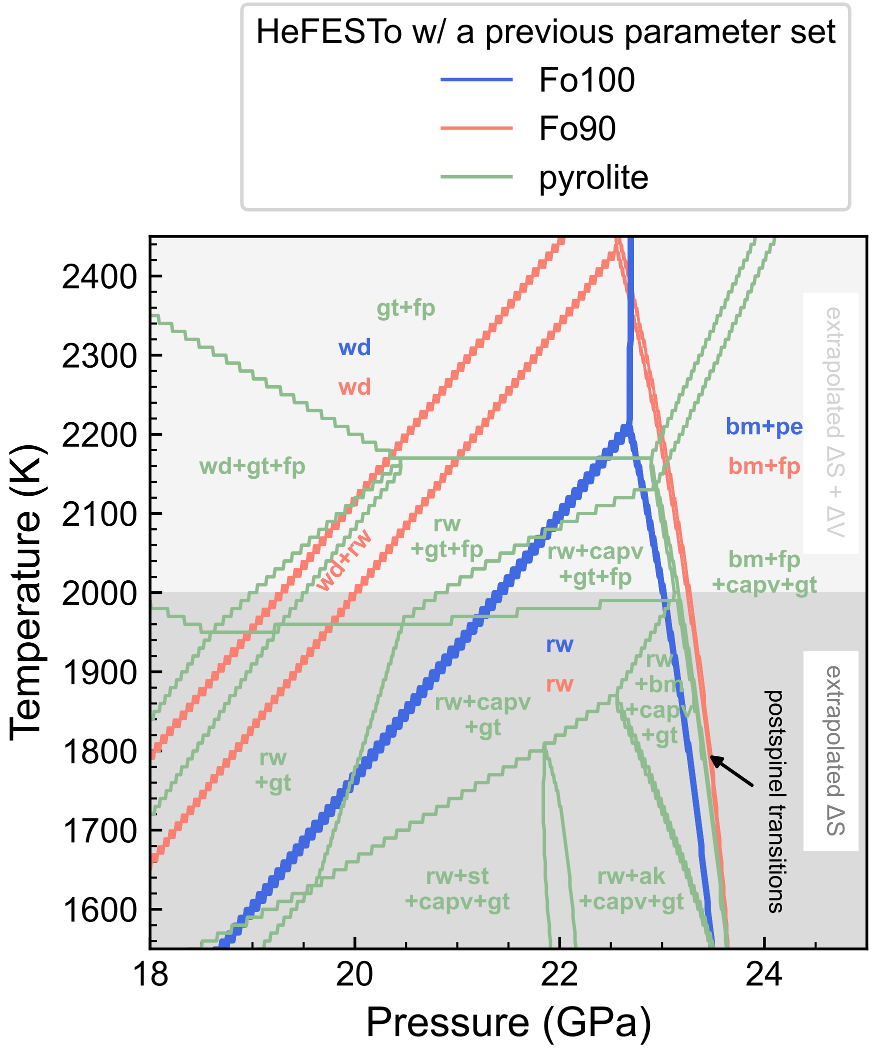

Fig. S1. Thermodynamic modeling of post-spinel transitions in Mg_2SiO_4, $(Mg_{0.9}Fe_{0.1})_2SiO_4$, and pyrolite. The modeled post-spinel phase transitions for Mg_2SiO_4, $(Mg_{0.9}Fe_{0.1})_2SiO_4$, and pyrolite, using the thermodynamic code HeFESTo (1, 2), are plotted in blue, red, and green, respectively. The Clapeyron slopes for Mg_2SiO_4 and pyrolite are nearly identical in the temperature range of 1400–2150 K, indicating that the post-spinel slope in Mg_2SiO_4 is a good proxy for that in mantle pyrolite. Differences in the P–T location of the transitions are minimal, with less than 0.5 GPa difference. To note, the pyrolite composition may undergo a different transition from ringwoodite (*rw*) to garnet (*gt*) and ferropericlase (*fp*) above 2150 K, which is not observed in Mg_2SiO_4 or $(Mg_{0.9}Fe_{0.1})_2SiO_4$. This *rw* ↔ *gt* + *fp* transition has been considered for the plumes in Figure 3. The gray areas indicate the P–T space where ΔS and ΔV are extrapolated without experimental validation. Since the Clapeyron slope is defined as ($\gamma = \frac{\partial P}{\partial T} = \frac{\Delta S}{\Delta V}$), it demonstrates the unreliability of thermodynamic models for accurate phase boundary predictions and highlights the need for direct experimental observations.

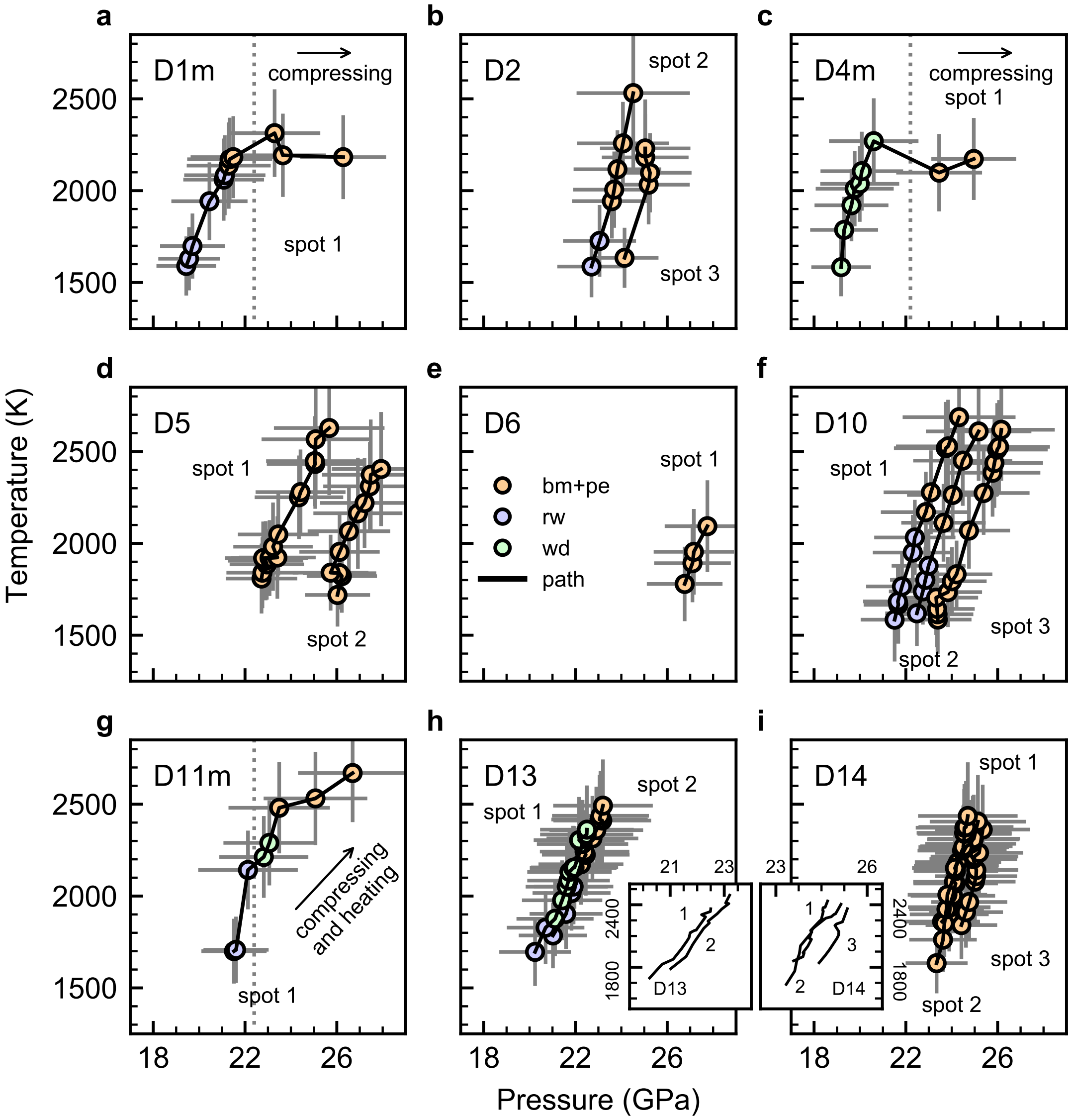

Fig. S2. In situ phase stability observations of Mg_2SiO_4 collected from LH-DAC experiments using synchrotron XRD. Panels **a–i** show the P–T conditions of various heated spots where XRD patterns were collected during experiments D1m, D2, D4m, D5, D6, D10, D11m, D13, and D14. For experiments D1m, D4m, and D11m, a membrane cell enabled simultaneous heating and compression. Solid black lines represent the P–T paths for phase stability observations in each run, with grey crosses indicating uncertainties in P and T (Dataset S1). Phases are color-coded: *wd* (green), *rw* (purple), and *bm+pe* (orange).

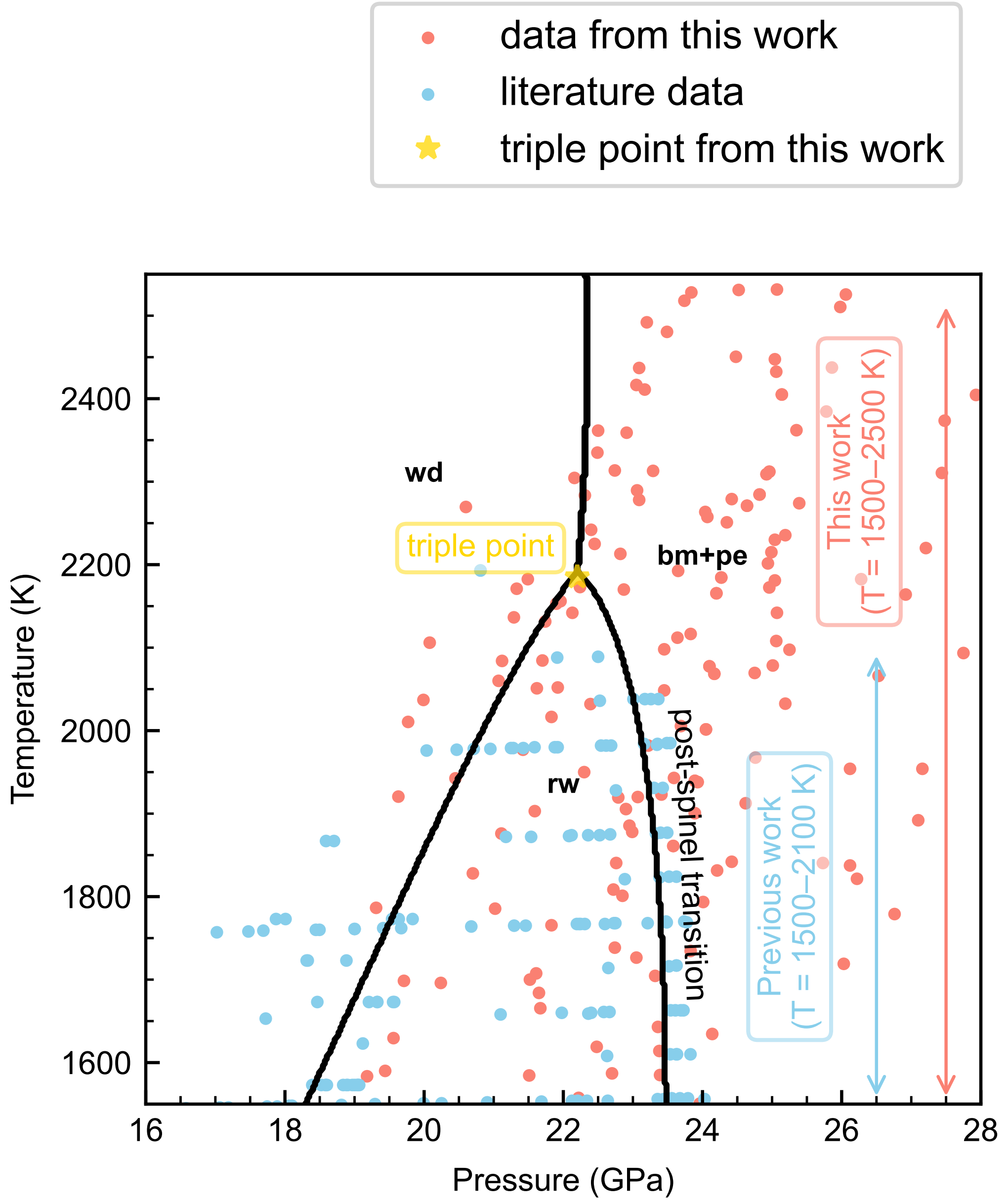

Fig. S3. Comparison between the P–T ranges investigated in previous experiments and in this work. The red shaded region represents the experimental P–T range covered in this study (1500 K to 2500 K), while the blue shaded region indicates the P–T range reported by previous work, including Chanyshev et al. (2022)(19), which is limited to 1500 K to 2100 K. The gold star marks the triple point derived from our analysis, which plays a critical role in constraining the shape and nonlinearity of the post-spinel boundary. By extending the temperature range beyond 2100 K, our data provide new constraints on the triple point, and combined with curated literature data, our analysis quantifies the nonlinearity of the post-spinel transition.

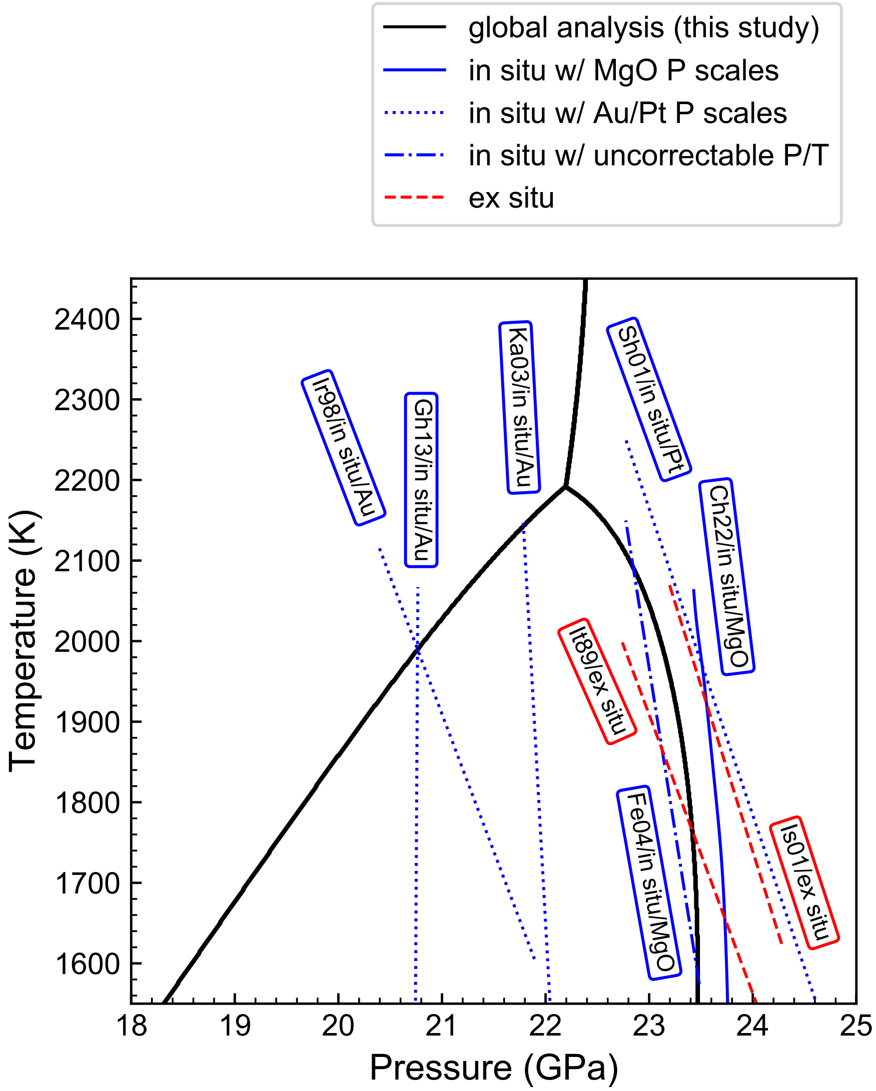

Fig. S4. Comparison of our machine-learned global analysis of the Mg_2SiO_4 phase diagram with post-spinel boundaries reported in the literature. The bold black lines represent our global analysis based on selected in situ MA experiments from the literature ([Ka04], [In06], [Ka09], [Ch22]) with recalculated P and T values, combined with our new in situ LH-DAC experiments. Excluded literature studies are represented by red dashed lines (ex situ experiments), blue dotted lines (in situ experiments using Au or Pt pressure standards), and blue dashed-dotted lines (experiments with uncorrectable P–T measurements).

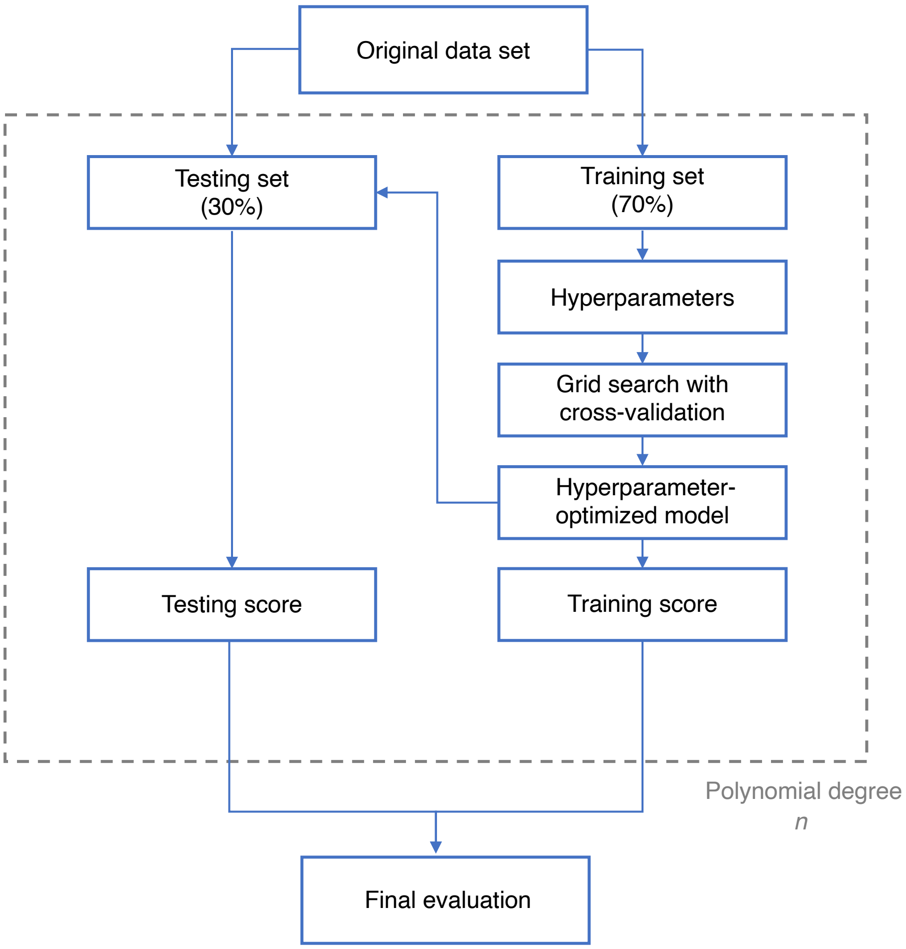

Fig. S5. Flow Chart of Supervised Learning Procedure. The original dataset is split into two sets: a training set (70%) and a testing set (30%). With the training dataset, hyperparameters in the multi-class logistic model are optimized using grid searching with cross-validation. The F_1 scores are estimated for the hyperparameter-optimized multi-class logistic models for polynomials of degree 1 to 10 on the train set and test set. The best model is selected based on the F_1 score of the testing set in the final evaluation.

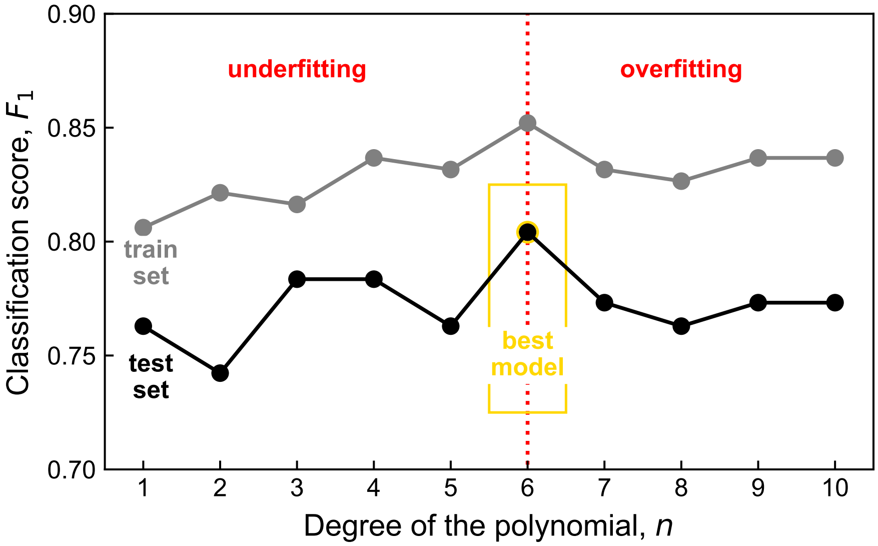

Fig. S6. Model performance (measured by F_1 score) as a function of log-odds/logit polynomial degree. Solid grey circles represent the train set while solid black circles denote the test set. The model's performance reaches its maximum at degree 6 for the test set, after which it declines. Of the polynomial degrees assessed here (n = 1–10), n = 6 (highlighted in gold) optimally balances the bias–variance trade-off. Note that this is the degree of the polynomial in the log-odds/logit function; the phase boundaries themselves are not described by these polynomials.

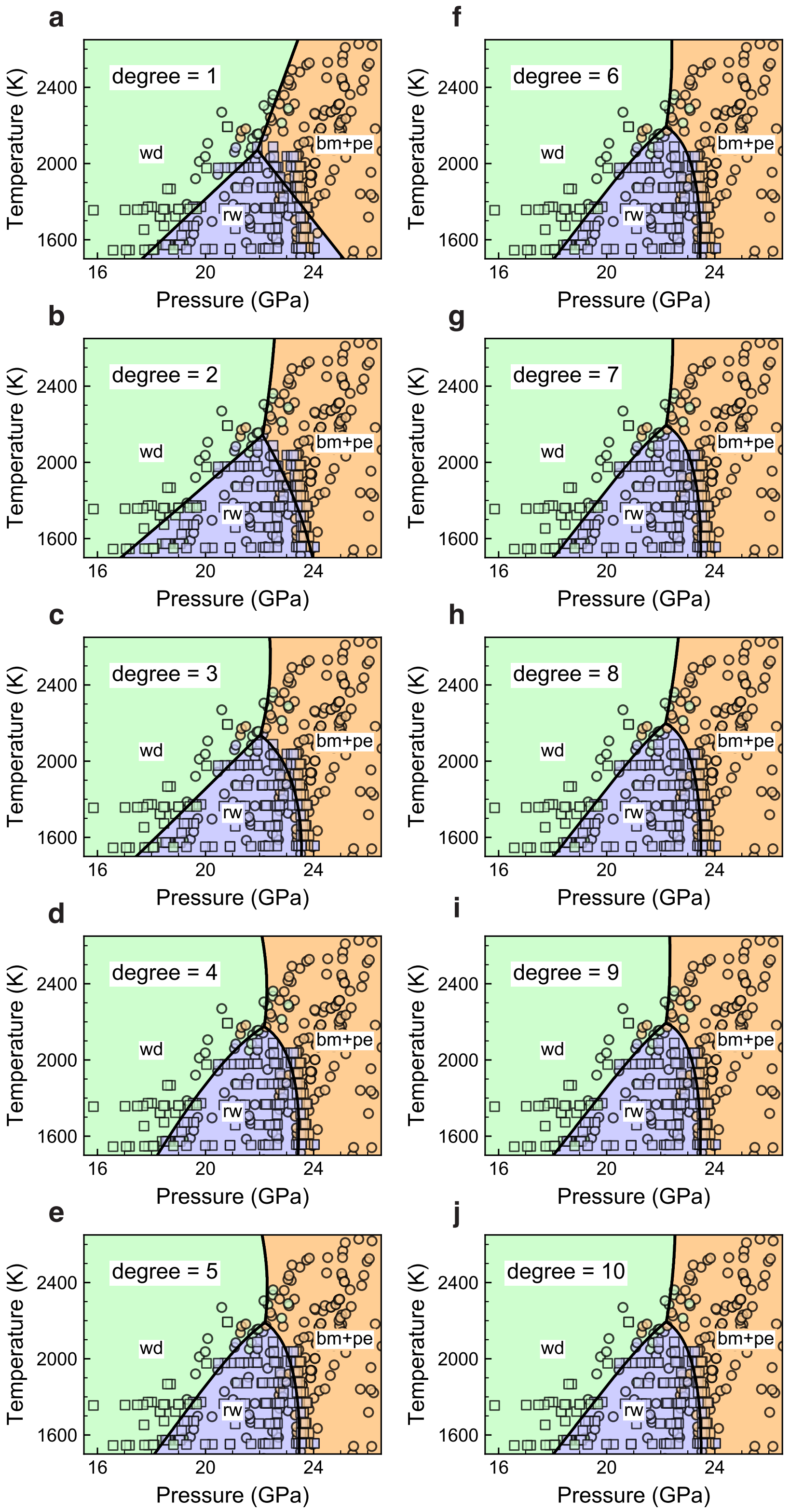

Fig. S7. Model selection for the Mg_2SiO_4 phase diagram. (a–j) Hyperparameter-optimized predictions for phase stability fields of wd, rw, and $bm{+}pe$ with different degree of log-odds/logit polynomials (n = 1–10) using a multi-class logistic model and supervised learning. Of the polynomial degrees assessed here, n = 6 (f) optimally balances the bias–variance trade-off. For n > 6, the phase diagram becomes stabilized, providing visual evidence that the model has converged. Symbols and colors are the same as in Figure 2.

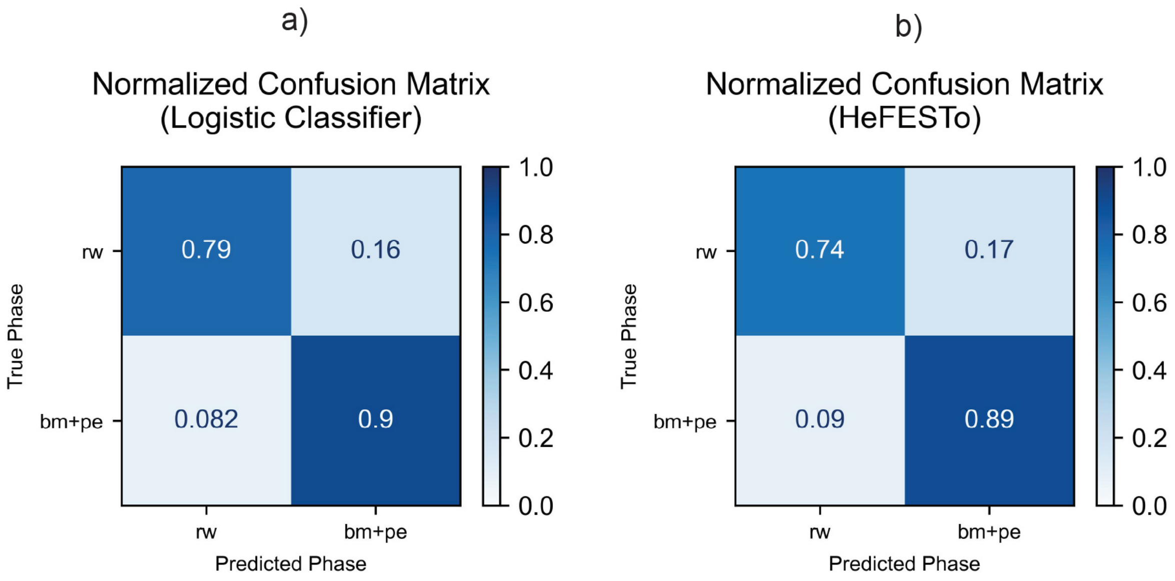

Fig. S8. Comparison of normalized confusion matrices for phase diagram models using (a) the logistic classifier (this work) and (b) the thermodynamic model (HeFESTo), evaluated for the post-spinel boundary between *rw* and *bm+pe*. The diagonal elements represent correctly predicted phases, while the off-diagonal elements indicate misclassified phases. The logistic classifier achieves higher scores for both phases, with an accuracy of 79% for *rw* and 90% for *bm+pe*, compared to 74% and 89% for HeFESTo, respectively.

References

1. L Stixrude, C Lithgow-Bertelloni, Thermodynamics of mantle minerals–I. Physical properties. *Geophys. J. Int.* **162**, 610–632 (2005).
2. L Stixrude, C Lithgow-Bertelloni, Thermodynamics of mantle minerals–II. Phase equilibria. *Geophys. J. Int.* **184**, 1180–1213 (2011).
3. H Kojitani, T Inoue, M Akaogi, Precise measurements of enthalpy of postspinel transition in mg2sio4 and application to the phase boundary calculation. *J. Geophys. Res. Solid Earth* **121**, 729–742 (2016).
4. Y Akahama, H Kawamura, Pressure calibration of diamond anvil Raman gauge to 310 GPa. *J. Appl. Phys.* **100** (2006).
5. A Dewaele, M Torrent, P Loubeyre, M Mezouar, Compression curves of transition metals in the mbar range: Experiments and projector augmented-wave calculations. *Phys. Rev. B* **78** (2008).
6. C Prescher, VB Prakapenka, DIOPTAS: A program for reduction of two-dimensional X-ray diffraction data and data exploration. *High Press. Res.* **35**, 223–230 (2015).
7. AJ Campbell, et al., High pressure effects on the iron–iron oxide and nickel–nickel oxide oxygen fugacity buffers. *Earth Planet. Sci. Lett.* **286**, 556–564 (2009).
8. TS Sokolova, PI Dorogokupets, AM Dymshits, BS Danilov, KD Litasov, Microsoft excel spreadsheets for calculation of P–V–T relations and thermodynamic properties from equations of state of MgO, diamond and nine metals as pressure markers in high-pressure and high-temperature experiments. *Comput. Geosci.* **94**, 162–169 (2016).
9. S Tateno, T Komabayashi, K Hirose, N Hirao, Y Ohishi, Static compression of B2 KCl to 230 GPa and its P–V–T equation of state. *Am. Mineral.* **104**, 718–723 (2019).
10. KD Litasov, et al., Thermal equation of state to 33.5 gpa and 1673 k and thermodynamic properties of tungsten. *J. Appl. Phys.* **113** (2013).
11. CR Harris, et al., Array programming with NumPy. *Nature* **585**, 357–362 (2020).
12. M Newville, et al., LMFIT: Non-linear least-square minimization and curve-fitting for Python. *Astrophys. Source Code Libr.* pp. ascl–1606 (2016).
13. C Ostrouchov, Peak fitting XRD data with Python [https://chrisostrouchov.com/post/peak_fit_xrd_python/] (2018).
14. CM Bishop, *Pattern Recognition and Machine Learning* eds. M Jordan, J Kleinberg, B Schölkopf. (Springer), (2006).
15. A Kavner, T Speed, R Jeanloz, Statistical analysis of phase-boundary observations. (Cambridge University Press), pp. 71–80 (2011).
16. G James, D Witten, T Hastie, R Tibshirani, *An Introduction to Statistical Learning* eds. G Casella, S Fienberg, I Olkin. (Springer), (2013).
17. F Pedregosa, et al., Scikit-learn: Machine learning in python. *J. Mach. Learn. Res.* **12**, 2825–2830 (2011).
18. J ZhangZhou, et al., Geochemistry : Automated machine learning python framework for tabular data. *Geochem. Geophys. Geosystems* **25**, e2023GC011324 (2024) e2023GC011324 2023GC011324.
19. A Chanyshev, et al., Depressed 660-km discontinuity caused by akimotoite–bridgmanite transition. *Nature* **601**, 69–73 (2022).
