# Supplementary material for: Nonlinearity of the post-spinel transition and its expression in slabs and plumes worldwide
Source: Nat Commun. 2025 Jan 26;16:1039. doi: 10.1038/s41467-025-56231-z (PMC11762276; doi:10.1038/s41467-025-56231-z)
Supplement: Supplementary file 3 — Description of Additional Supplementary Files [file 41467_2025_56231_MOESM3_ESM.pdf]

Description of Additional Supplementary Files

Supplementary Data 1: New in situ LH-DAC experimental dataset on the Mg2SiO4 phase diagram.

Supplementary Data 2: In situ phase stability observations on Mg2SiO4 compiled from literature with corrected pressures and temperatures.

Supplementary Data 3: In situ phase stability observations on Mg2SiO4 compiled from literature with uncorrected pressures and temperatures.

Supplementary Data 4: Subducting slabs and their T660 and γpost-spinel.

Supplementary Data 5: Hotspot-associated plumes and their T660 and γpost-spinel.

Supplementary Data 6: Model selection results.

Supplementary Data 7: Code to construct a globally optimized phase diagram using multi-class regression and supervised learning.
