# Supplementary figures and images for: Nonlinearity of the post-spinel transition and its expression in slabs and plumes worldwide
Source: Nat Commun. 2025 Jan 26;16:1039. doi: 10.1038/s41467-025-56231-z (PMC11762276; doi:10.1038/s41467-025-56231-z)

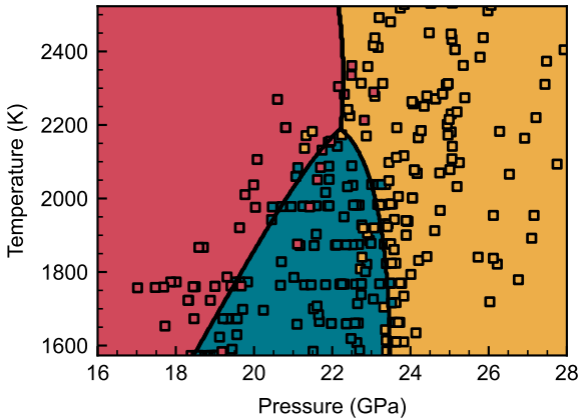

Supplement: Supplementary file 10 — Supplementary Data 7 [file 41467_2025_56231_MOESM10_ESM.zip › MLPD-main/logit_reg_fit.pdf]
